# Rift Valley Fever Virus Infects the Posterior Segment of the Eye and Induces Inflammation in a Rat Model of Ocular Disease

**DOI:** 10.1128/jvi.01112-22

**Published:** 2022-10-04

**Authors:** Madeline M. Schwarz, Kaleigh A. Connors, Katherine A. Davoli, Cynthia M. McMillen, Joseph R. Albe, Ryan M. Hoehl, Matthew J. Demers, Safder S. Ganaie, David A. Price, Daisy W. Leung, Gaya K. Amarasinghe, Anita K. McElroy, Douglas S. Reed, Amy L. Hartman

**Affiliations:** a Center for Vaccine Research, University of Pittsburgh, Pittsburgh, Pennsylvania, USA; b Department of Infectious Diseases and Microbiology, School of Public Health, University of Pittsburgh, Pittsburgh, Pennsylvania, USA; c Ophthalmic and Visual Sciences Research Center, Department of Ophthalmology, University of Pittsburgh School of Medicine, Pittsburgh, Pennsylvania, USA; d Department of Pathology and Immunology, Washington University School of Medicine in St. Louis, St. Louis, Missouri, USA; e Department of Medicine, Washington University School of Medicine in St. Louis, St. Louis, Missouri, USA; f Division of Pediatric Infectious Disease, Department of Pediatrics, University of Pittsburgh, Pittsburgh, Pennsylvania, USA; Instituto de Biotecnologia/UNAM

**Keywords:** Rift Valley fever, aerosol, bunyavirus, eye disease, ocular disease, retinitis, uveitis

## Abstract

People infected with the mosquito-borne Rift Valley fever virus (RVFV) can suffer from eye-related problems resulting in ongoing vision issues or even permanent blindness. Despite ocular disease being the most frequently reported severe outcome, it is vastly understudied compared to other disease outcomes caused by RVFV. Ocular manifestations of RVFV include blurred vision, uveitis, and retinitis. When an infected individual develops macular or paramacular lesions, there is a 50% chance of permanent vision loss in one or both eyes. The cause of blinding ocular pathology remains unknown in part due to the lack of a tractable animal model. Using 3 relevant exposure routes, both subcutaneous (SC) and aerosol inoculation of Sprague Dawley rats led to RVFV infection of the eye. Surprisingly, direct inoculation of the conjunctiva did not result in successful ocular infection. The posterior segment of the eye, including the optic nerve, choroid, ciliary body, and retina, were all positive for RVFV antigen in SC-infected rats, and live virus was isolated from the eyes. Proinflammatory cytokines and increased leukocyte counts were also found in the eyes of infected rats. Additionally, human ocular cell lines were permissive for Lrp1-dependent RVFV infection. This study experimentally defines viral tropism of RVFV in the posterior segment of the rat eye and characterizes virally-mediated ocular inflammation, providing a foundation for evaluation of vaccines and therapeutics to protect against adverse ocular outcomes.

**IMPORTANCE** Rift Valley fever virus (RVFV) infection leads to eye damage in humans in up to 10% of reported cases. Permanent blindness occurs in 50% of individuals with significant retinal scarring. Despite the prevalence and severity of this outcome, very little is known about the mechanisms of pathogenesis. We addressed this gap by developing a rodent model of ocular disease. Subcutaneous infection of Sprague Dawley rats resulted in infection of the uvea, retina, and optic nerve along with the induction of inflammation within the posterior eye. Infection of human ocular cells induced inflammatory responses and required host entry factors for RVFV infection similar to rodents. This work provides evidence of how RVFV infects the eye, and this information can be applied to help mitigate the devastating outcomes of RVF ocular disease through vaccines or treatments.

## INTRODUCTION

Rift Valley fever virus (RVFV) is an emerging threat to both human and animal health (1). Originally identified in the Rift Valley of Kenya, RVFV (Order *Bunyavirales*; Family *Phenuiviridae*) is a virus of pandemic potential found in Africa, the Saudi Arabian Peninsula, and Madagascar (2–5) for which there are no FDA-approved human vaccines or effective post-exposure therapeutic treatments. RVFV is lethal in livestock and infects people either through direct contact with animals or by mosquito bite. In humans, RVF most commonly manifests as febrile illness with flu-like symptoms. While most individuals recover from this self-limited disease, a subset progress to more severe clinical outcomes such as hepatitis, hemorrhagic fever, encephalitis, or ocular disease, which is the most common complication (6–8).

Ocular manifestations of human RVF have been described in the literature since the 1950s, yet very little has been done to experimentally dissect the pathogenesis leading to this outcome. Case reports issued during the large 1977 Egyptian outbreak found that patients experienced retro-orbital pain, joint pain, headaches, and recurring fevers, with bilateral macular or paramacular lesions that were visualized by fundus imaging, some of which were described as having a “vitreous haze” (9). This could indicate cellular infiltration into the vitreous humor following infection. In these initial case reports, some patients completely resolved the retinal lesions and vision improved, while others retained extensive retinal scarring resulting in permanent vision loss (9).

A larger cohort of individuals were assessed during the Saudi Arabia outbreak in 2000. Fifteen percent of 206 hospitalized individuals with severe forms of RVF had concurrent ocular lesions (10). Intriguingly, over 50% of outpatient individuals sought medical attention because of significant vision-related issues such as uveitis, optic disc edema, and retinal hemorrhage and/or lesions (10). Vision only improved in 13% of these outpatient cases; in fact, 71% of the eyes assessed were considered legally blind (10). More recently, an individual from Sudan with RVF had extensive optic nerve atrophy and retinitis contributing to complete vision loss in the right eye, and both retinitis and retinal pigment epithelial thickening contributed to visual impairment in the left eye (11). Therefore, ocular disease resulting from RVFV infection is a serious, sometimes debilitating outcome which mainly affects the posterior portion of the eye, in particular the retina.

While some animal studies have noted virus or viral RNA in the eyes of experimentally infected animals (12–14), the pathogenic mechanisms remain unexplored. In a prior rodent study, uveitis was observed in 3/82 rats after aerosol challenge with RVFV following immunization with TSI-GSD-200, a formalin-inactivated RVFV vaccine (15). Ocular involvement was not investigated further following this observation. Here, we use Sprague Dawley rats to understand the pathogenesis of ocular RVFV infection. We evaluated different infection routes and found that subcutaneous (SC) inoculation with RVFV resulted in efficient eye infection, while direct conjunctival exposure did not. Like the documented human cases, posterior structures of the eye such as the uvea, optic nerve, and retina were infected by RVFV. Infiltrating leukocytes and increased levels of cytokines/chemokines were also noted in the eyes following SC RVFV infection. Human ocular cells were highly permissive to RVFV in an Lrp1-dependent manner and induced an inflammatory response. These findings provide a detailed experimental investigation into RVF ocular disease and highlight the potential use of this rodent model for evaluation of the efficacy of vaccines and therapeutics for prevention or treatment of RVF eye infection.

## RESULTS

### Subcutaneous and aerosol inoculation of rats with RVFV results in efficient infection of ocular tissues.

**FIG 1:**
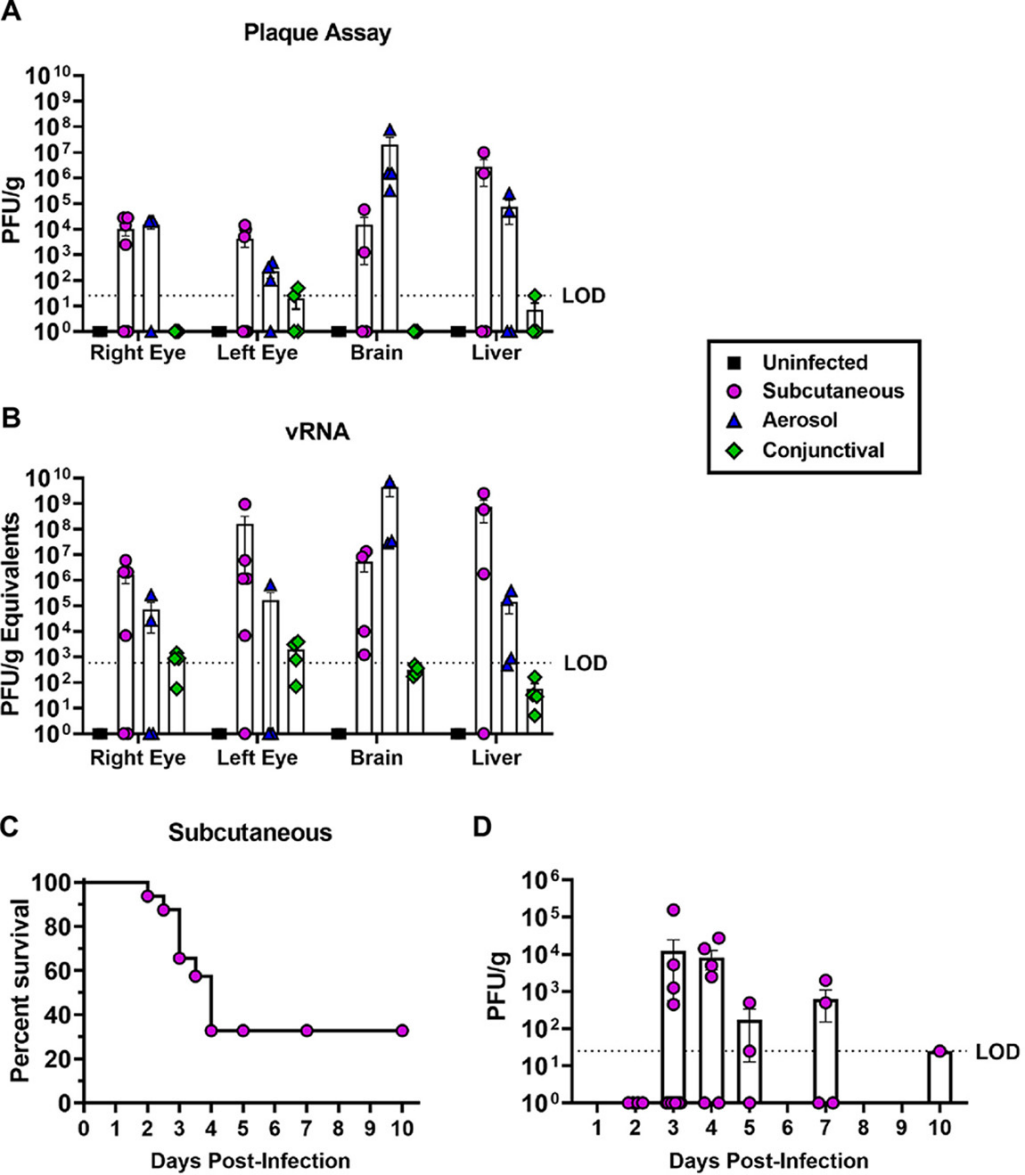
RVFV ZH501 titers in the Sprague Dawley rat eye following subcutaneous, aerosol, or conjunctival inoculation. (A) Infectious virus or (B) viral RNA (vRNA) in whole eye homogenates of Sprague Dawley rats at 4 dpi following sham (uninfected), subcutaneous, aerosol, or conjunctiva inoculation with 1000 PFU RVFV ZH501. (C) Survival of Sprague Dawley rats following subcutaneous inoculation with 1000 PFU of RVFV ZH501. (D) Infectious virus in the right and left whole eye homogenates of rats infected subcutaneously with ~1000 PFU of RVFV ZH501 and euthanized between 2 and 10 dpi. Each data point represents one eye from one rat across three experiments. Error bars represent standard error mean. LOD = Limit of detection.

Previous studies have detected RVFV antigen and viral RNA in the eyes of infected rodents (12, 13), yet a direct comparison of ocular tropism between different infection routes had not been tested. Therefore, we evaluated 3 clinically relevant routes of inoculation to compare efficiency of eye infection. Sprague Dawley rats were inoculated with ~1000 PFU of RVFV (pathogenic BSL-3 strain ZH501) either subcutaneously (SC) to represent peripheral infection (*n* = 6 animals), by exposure to small particle aerosol to recapitulate inhalational infection (*n* = 4 animals), or through deposition on the conjunctiva to mimic direct ocular mucosal exposure (*n* = 4 animals). At 4 days postinfection (dpi), the rats were euthanized, perfused with saline, and both eyes, brain, and liver were collected. Homogenized tissues were analyzed by viral plaque assay (VPA) for infectious titers and q-RT-PCR for viral RNA which revealed that both SC and aerosol infection resulted in moderate levels of infectious virus in the eye (10^3^-10^4^ PFU/g tissue) at this time point (Fig. 1A and B). Not all RVFV-infected rats developed ocular infection (Fig. 1A); approximately 50% of SC and 62% of aerosol-infected rats had infectious virus in the eye at 4 dpi. Additionally, one SC-infected rat had unilateral ocular infection while 3 rats had bilateral. All aerosol-infected rats with infectious virus in their eyes had bilateral ocular infection. Surprisingly, direct inoculation of the conjunctiva with RVFV did not result in detectable titers in the eye at 4 dpi or clinical signs of illness (Fig. 1A). Based on these initial experiments, all subsequent experiments utilized SC inoculation as it resulted in reproducible infection of the eye.

In further experiments, SC inoculation of rats with 1000 PFU of RVFV strain ZH501 resulted in ~30% survival past 4 dpi (Fig. 1C). To determine when RVFV titers peak in the eye following SC infection, another cohort of rats were inoculated with RVFV and underwent planned euthanasia at 2, 3, 4, 5, 7, or 10 dpi. Infectious virus in the eyes peaked between 3 and 4 dpi but moderate levels could be detected out to 7 dpi in surviving animals (Fig. 1D). In this experiment, the right eye was fixed for histological analysis, therefore we were unable to determine the frequency of unilateral or bilateral ocular infection.

### RVFV infection promotes a proinflammatory environment in the eye.

**FIG 2:**
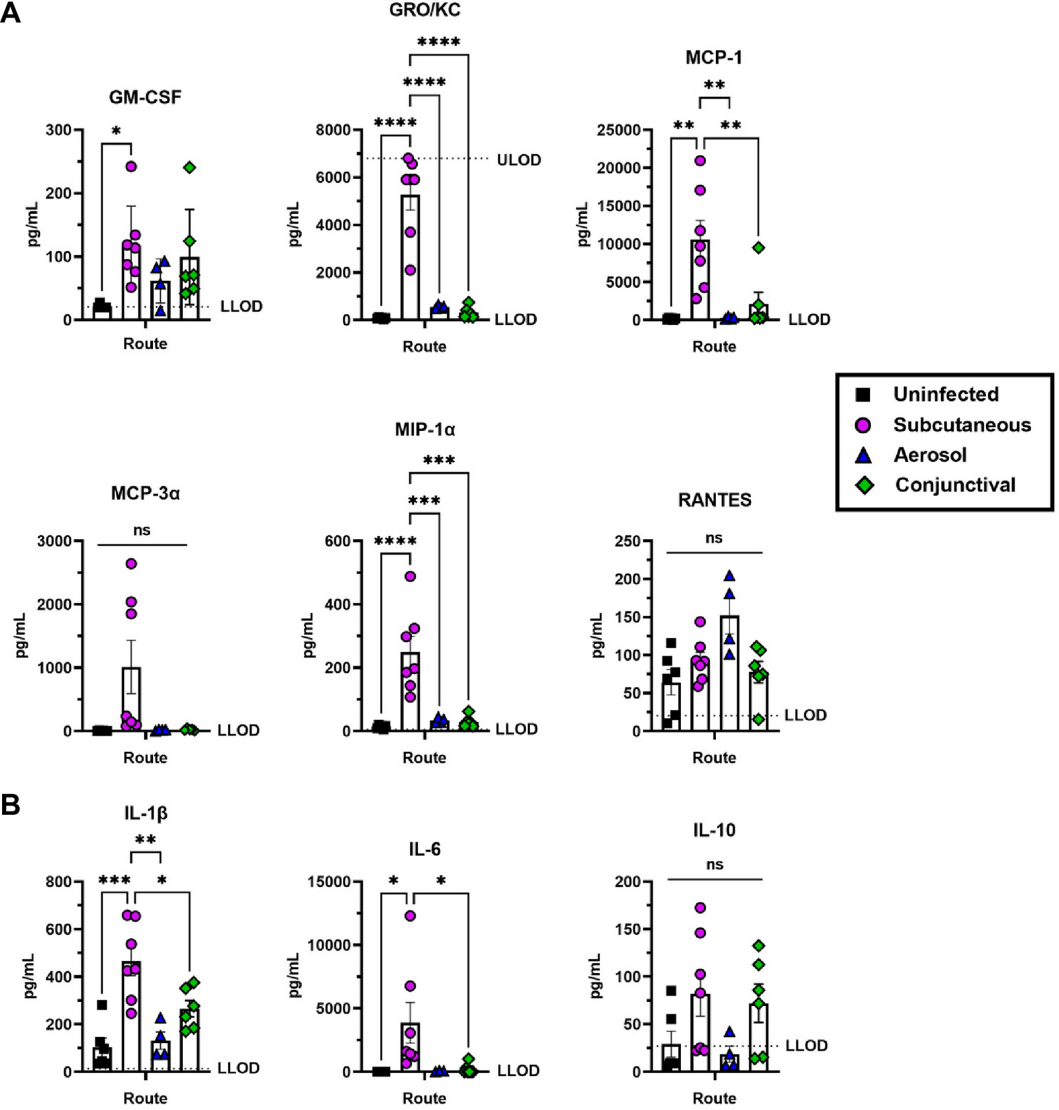
Chemokines and proinflammatory cytokines expressed in the eye following RVFV ZH501 infection of Sprague Dawley rats. (A) Chemokines or (B) proinflammatory cytokines detected in whole eye homogenates at 4 dpi of rats infected with 1000 PFU of RVFV ZH501 through sham (uninfected), subcutaneous, aerosol, or conjunctiva inoculation. Each data point represents one eye from one rat across four experiments. Error bars are standard error mean. LLOD = Lower limit of detection. ULOD = Upper limit of detection. Statistics were determined by one-way ANOVA. *, *P* < 0.05; **, *P* < 0.01; ***, *P* < 0.001; ****, *P* < 0.0001.

We evaluated the inflammatory environment of the eyes at 4 dpi using a Bio-Rad multiplex assay on eye homogenates obtained from SC, aerosol, or conjunctiva inoculated animals. Uninfected animals were sham inoculated conjunctively with growth media to control for a local inflammatory response from this inoculation technique. SC infection resulted in higher levels of inflammatory mediators than the other infection routes (Fig. 2). The chemokine GM-CSF was expressed at significantly higher levels in eyes from SC-infected rats compared to uninfected controls (Fig. 2A). Chemokines GRO/KC, MCP-1 and MIP-1α, as well as proinflammatory cytokines IL-1β and IL-6, were highly expressed in eyes of SC-infected rats compared to both uninfected controls and aerosol- and conjunctiva-infected rat eyes (Fig. 2A and B). Although infectious virus and viral RNA were not detected in conjunctiva-infected rat eyes (Fig. 1A and B), there is an increase in proinflammatory factors in the whole eye homogenate compared to sham-infected rats based on the upregulation of GM-CSF and IL-1β, but these differences were not statistically significant (Fig. 2A and B). Levels of IL-10 were not significantly different between groups but were increased in both the SC and conjunctiva-infected rats (Fig. 2B). Additional factors analyzed in the multiplex are included in Fig. S1.

### RVFV infects posterior ocular structures.

**FIG 3:**
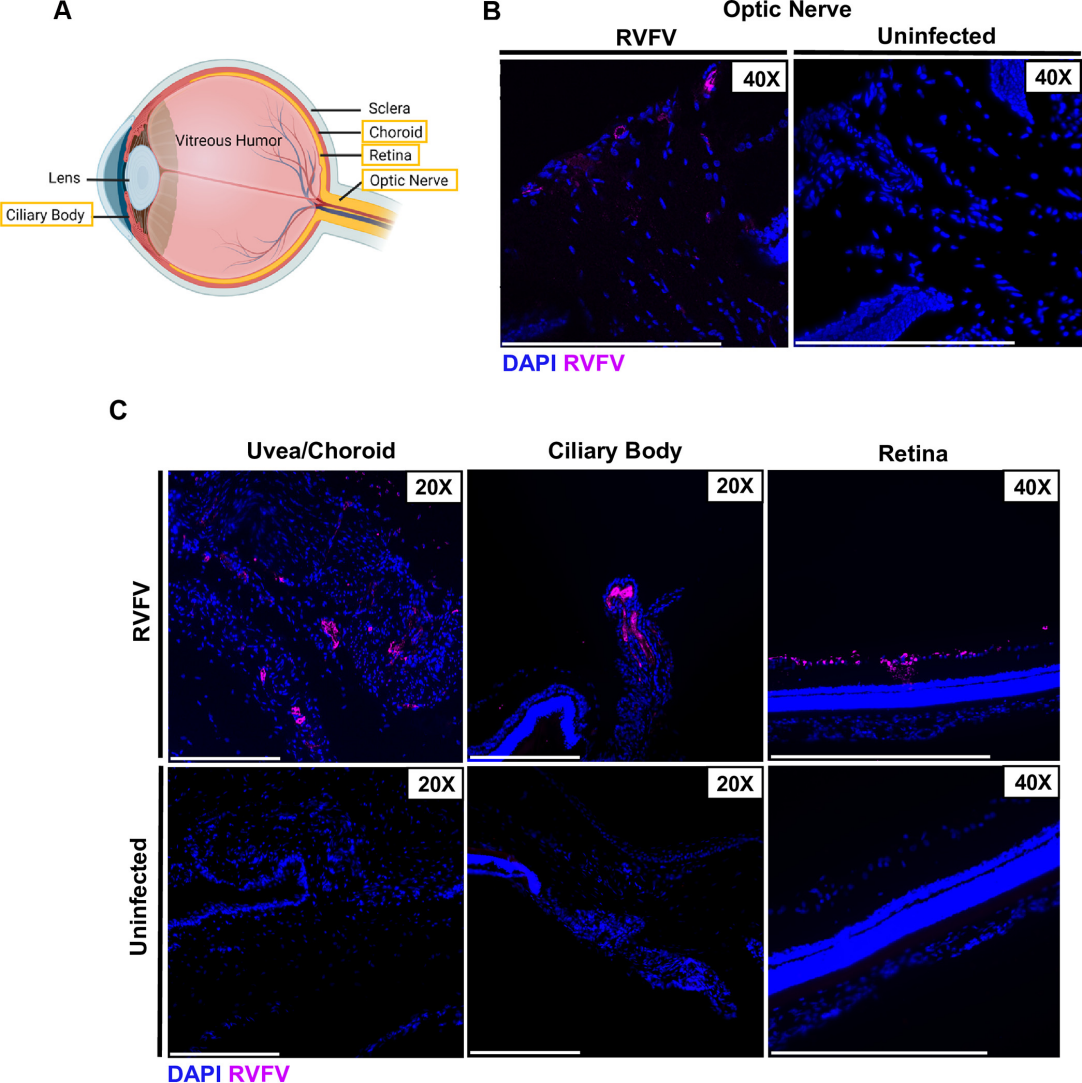
Tissue tropism of RVFV in the posterior portion of the eye at 3 dpi. (A) Schematic of ocular anatomy with structures of interest boxed in yellow. Created in BioRender.com. (B) Eyes were enucleated from Sprague Dawley rats 3 days following subcutaneous RVFV infection. RVFV antigen staining was found on the optic nerve head using an anti-RVFV N antibody (magenta). (C) RVFV was also found in the uvea, ciliary body, and ganglion layer of the retina at 3 dpi. Scale bar is 250 μm.

While human case reports of RVF ocular disease describe uveitis and retinitis, it remains unknown which ocular structures are directly infected by RVFV. The posterior portion of the eye is comprised of the optic nerve, the uvea, and the retina (Fig. 3A). The uvea is made up of the choroid and ciliary body which is included in both the anterior and posterior portion of the eye. Using fluorescence microscopy on eyes from SC-infected rats, we were able to detect RVFV infection in many of these structures at 3 dpi, including the optic nerve, uvea, ciliary body, and retina (Fig. 3B and C). The uvea is essential for regulating nutrient uptake and waste removal to and from the retina, and inflammation of this structure (uveitis) is commonly described in case reports of human RVF ocular disease (10, 16). The ciliary body is responsible for manually contracting the lens and regulating fluid in the anterior portion of the eye (17). RVFV staining was found in the ganglion layer of the retina, which is made up of irreplaceable neurons that send visual information to the optic nerve (Fig. 3C). These viral targets likely all play a role in the clinical outcomes of RVF ocular disease.

### Leukocytes infiltrate the posterior eye following RVFV infection.

**FIG 4:**
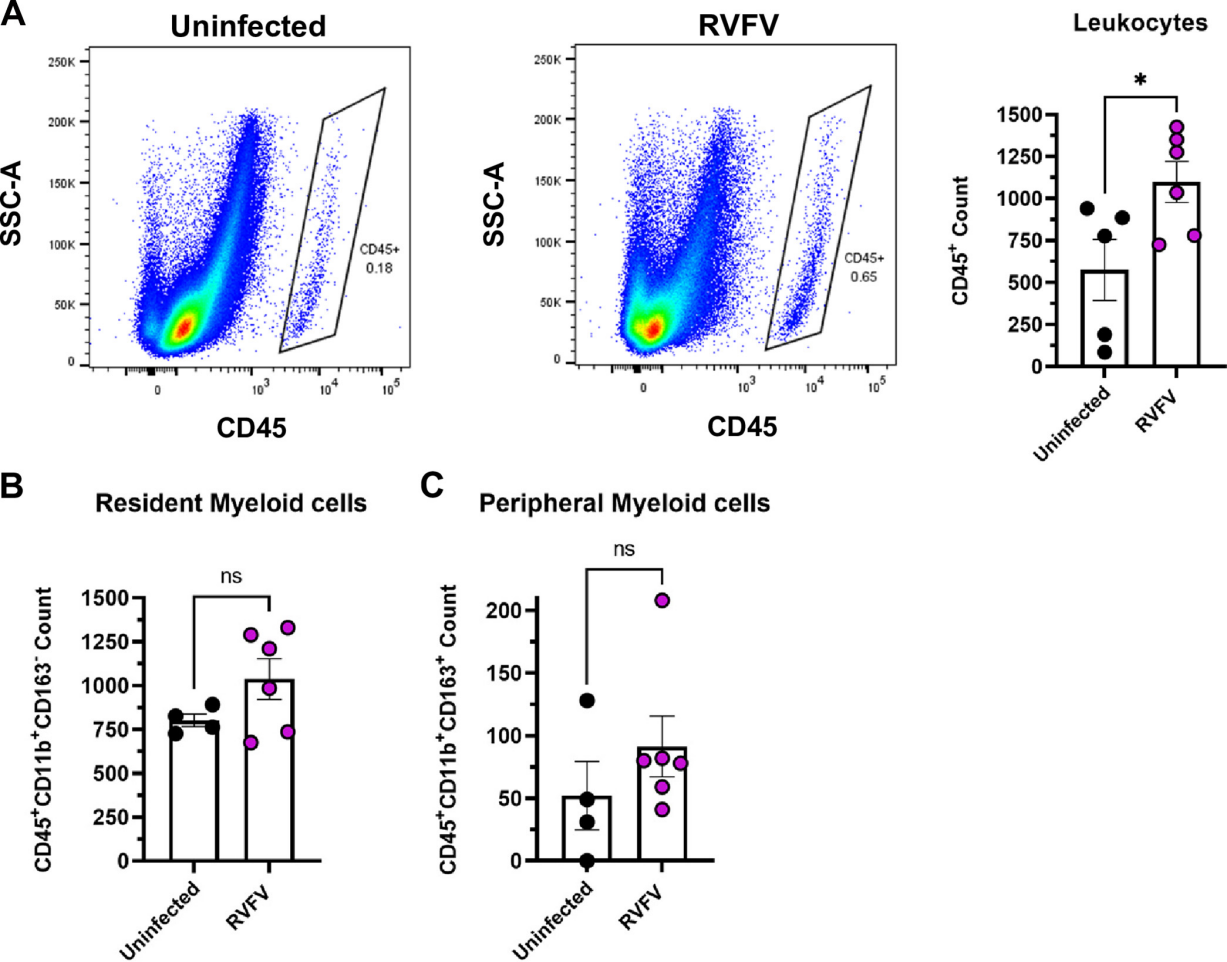
Significant increase in leukocyte counts in the posterior portion of RVFV-infected eyes. (A) CD45^+^ at 3 dpi in the right eye of rats (*n* = 6) infected SC with 1000 PFU of RVFV compared to uninfected (*n* = 5) rat eyes. (B) Resident myeloid cells (CD45^+^Cd11b^+^CD163^−^) and (C) peripheral myeloid cells (CD45^+^Cd11b^+^CD163^+^) in the posterior eyes of RVFV-infected rats compared (*n* = 4) to uninfected controls (*n* = 6). Each data point represents the right eye of one rat across two experiments. Error bars represent standard error mean. Statistics were determined by *unpaired t test*. *, *P* < 0.05.

Significant macrophage infiltration into the brain has been noted in a rat model of RVF encephalitis (18). To determine levels of immune cell infiltrates into the eye, rats were euthanized at 3 dpi and the right eye was enucleated for analysis. The posterior portion of the right eye was dissected and digested into a single cell suspension for flow cytometry analysis to measure inflammatory cell counts using CD45, CD11b, and CD163 as markers of immune cells (Fig. S2). Although not every rat develops RVFV infection in the eye following SC infection, the right eye from all RVFV-infected and uninfected rats were compared (Fig. 4). An equal number of events (5x10^5^) were collected from each eye sample to compare immune cell populations between infected and uninfected rats. Live cells were gated on CD45^+^ events, followed by CD11b and CD163 to assess resident (CD11b^+^CD163-) and infiltrating (CD11b^+^CD163^+^) myeloid cells (Fig. S2). Rats that were infected by SC inoculation had significantly more CD45^+^ cells present in the posterior portion of their eye at 3 dpi compared to uninfected controls (Fig. 4A). Additionally, SC-infected rats had increased counts of both resident myeloid cells and peripheral myeloid cells (Fig. 4B and C). There were very few CD45^+^CD11b- cells identified, indicating that lymphocytes were not a major component of the observed inflammation at 3 dpi.

### RVFV can infect human and bovine ocular cells.

**FIG 5:**
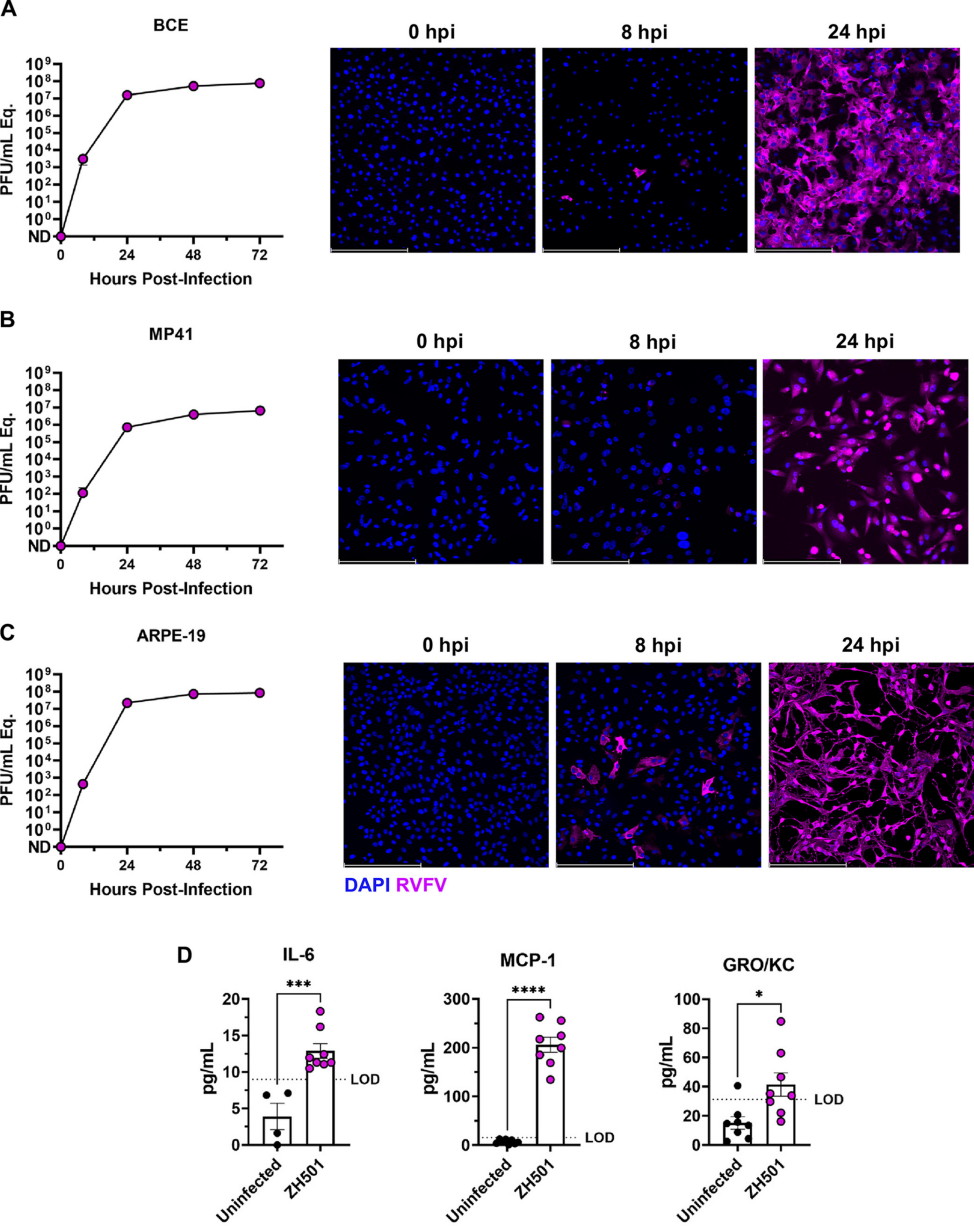
RVFV infection of bovine and human ocular cell lines induces proinflammatory factors. (A) Bovine corneal endothelial cell line BCE, (B) human uveal cell line MP41, and (C) human retinal pigment epithelial cell line ARPE-19 were infected with RVFV ZH501 at MOI 0.1. Supernatant samples were taken at 0, 8, 24, 48, and 72 hpi and analyzed by qRTPCR. Fluorescent images associated with each cell line depict RVFV ZH501 infection at 0, 8, and 24 hpi (20X). (D) IL-6, MCP-1, and GRO/KC protein were detected in the supernatant of RVFV ZH501-infected ARPE-19 cells at 24 hpi compared to uninfected controls. Data are representative of 2 experiments. Error bars are standard error mean. Scale bar is 250 μm. Statistics were determined by *unpaired t test*. *, *P* < 0.05; ***, *P* < 0.001; ****, *P* < 0.0001.

To determine if the ocular cell tropism observed in this rodent model translates to human and bovine ocular-derived cells, we infected human retinal pigment epithelial cells (ARPE-19), human uveal cells (MP41), and bovine corneal endothelial (BCE) cells with RVFV ZH501. Using a multiplicity of infection (MOI) of 0.1, all 3 cell lines were highly permissive to RVFV infection, with virus detected by 8 h postinfection (hpi) including extensive visualization of RVFV staining at 24 hpi (Fig. 5A to C).

Following the validation of the permissiveness of ARPE-19 cells to RVFV ZH501 (Fig. 5C), we sought to evaluate cytokine secretion. ARPE-19 cells were chosen for these studies because of the vital role these cells play in maintaining blood-retinal barrier integrity. Disruption or damage of the retinal epithelial cell layer could lead to infiltration of inflammatory cells into the retina causing devastating injury. ARPE-19 cells were infected with RVFV ZH501 at MOI of 0.1, and supernatant samples were collected at 24 hpi. Supernatants from infected and uninfected cells were analyzed for IL-6, MCP-1, and GRO/KC protein, as these factors were significantly upregulated in the whole eye homogenate in the rat model (Fig. 2). We found that all 3 factors were also significantly increased in the supernatant of infected ARPE-19 cells compared to uninfected controls (Fig. 5D), although the overall level of cytokine secretion was rather low compared to the *in vivo* data (Fig. 2). This supports that RVFV can infect human ocular cells that comprise structures like the retinal pigment epithelium and uvea that are essential for ensuring ocular integrity.

### RVFV infection of ARPE-19 cells involves Lrp1.

**FIG 6:**
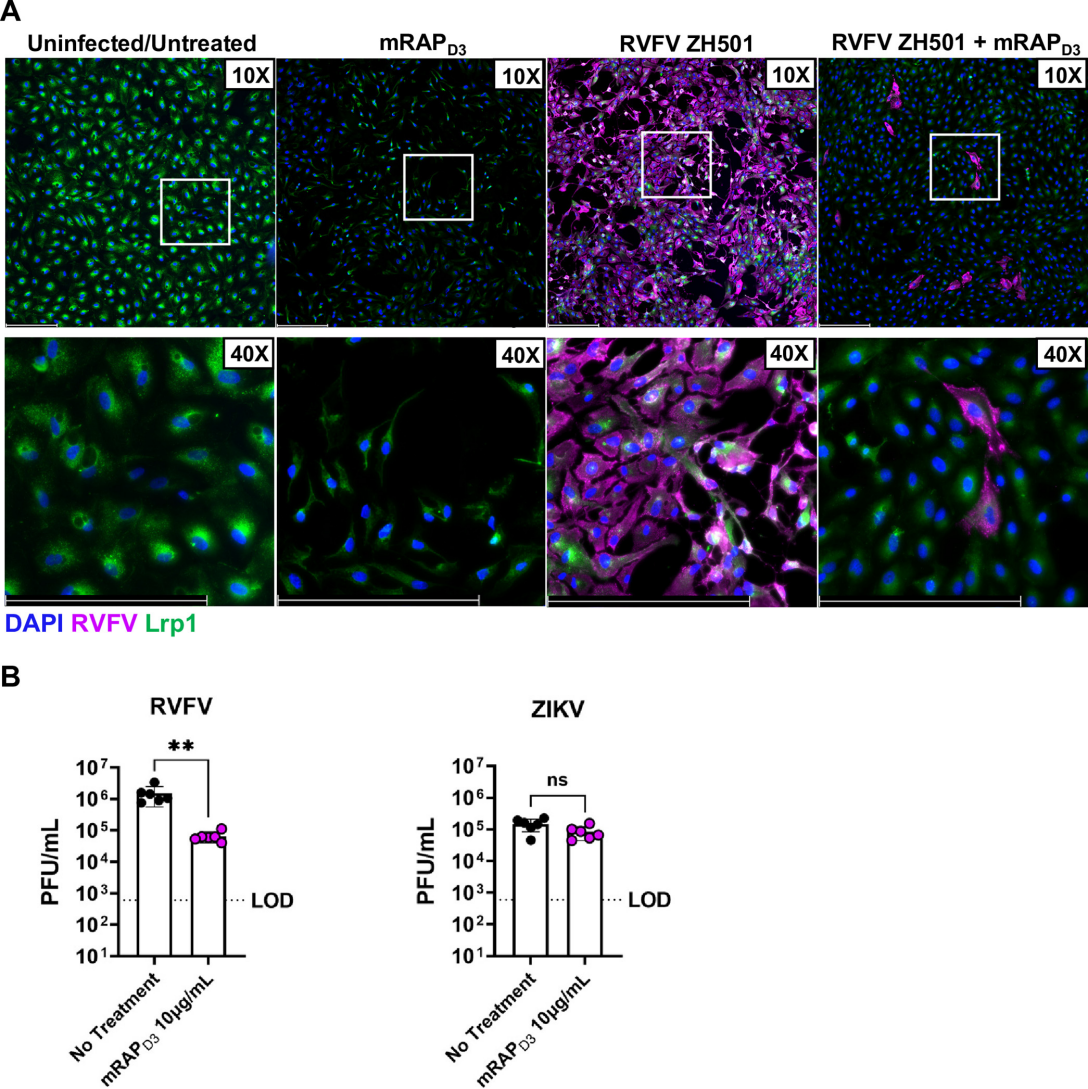
Pre-treatment of ARPE-19 cells with the Lrp1-binding agent RAP reduces RVFV ZH501 infection. (A) ARPE-19 cells were treated with 10 μg/mL of RAP, a high-affinity Lrp1 ligand, 1 h prior to infection with MOI 0.1 of RVFV ZH501 and stained for RVFV N (magenta) and Lrp1 (green) at 24 hpi. Scale bar is 250 μm. (B) ARPE-19 cells with or without RAP pre-treatment were infected with MOI 0.1 of RVFV ZH501 or ZIKV as a control. Samples were collected at 24 hpi for RVFV and 48 hpi for ZIKV and analyzed by viral plaque assay. Data are representative of 2 experiments. Error bars are standard error mean. Statistics were determined by *unpaired t test*. **, *P* < 0.01.

Low density lipoprotein receptor (LDLR)-related protein 1 (Lrp1) was recently identified as a receptor for RVFV (19). Although this was validated in cell lines of taxonomically different species, including BCE cells, the role of Lrp1 in human ocular infection is unclear. To determine if Lrp1 may play a role in RVFV infection of human ocular cells, we used a high-affinity Lrp1 ligand, the receptor associated protein (RAP), which blocks RVFV infection of other cell types (19). ARPE-19 cells were pre-treated with mouse RAP domain 3 (mRAP_D3_) prior to RVFV ZH501 infection. RAP treatment significantly reduced RVFV infection of these cells as visualized by ICC (Fig. 6A). Infectious RVFV was also significantly reduced in the supernatant of mRAP_D3_-treated cells compared to untreated cells (Fig. 6B). This observation was specific to RVFV, as there was no significant reduction in controls studies with Zika virus (ZIKV) following similar mRAP_D3_ treatment (Fig. 6B). These studies indicate that Lrp1 may play a role in efficient RVFV infection of human ocular cells.

## DISCUSSION

Humans in RVFV-endemic areas are at risk of acquiring RVFV infection through direct mosquito bite, inhalation of aerosolized particles during birthing and butchering of livestock, or direct mucosal exposure to infected animal bodily secretions or tissues (7, 20, 21). It is unknown whether a particular infection route is more likely to result in ocular infection and subsequent disease. Each of these routes were considered as potential mechanisms for eye infection during the development of this rodent model. While SC and aerosol inoculation both resulted in detectable virus in the eye, it was somewhat variable in that not every infected rat had virus in the eye, and some rats had disparate levels of virus in the 2 eyes. In addition, activation of the innate inflammatory response clearly differed between the 2 routes based on the cytokines and chemokines detected in the eye, where SC-infected animals had significantly more inflammatory factors than aerosol-infected rats. These immunological differences could be due to SC infection igniting a strong peripheral immune response, whereas aerosol infection results in direct infection of the olfactory epithelium and olfactory bulb (18).

Most surprising was the finding that direct conjunctival inoculation did not result in either systemic infection or infection of ocular structures. Prior to these studies, we hypothesized that direct ocular mucosal exposures during animal birthing or butchering of infected animals may significantly contribute to human infection burden; however, we discovered that this is a remarkably inefficient mechanism of infection, at least in rats. We directly instilled viral inoculum into the conjunctiva of each animal, and it is likely that the natural tear barrier of the eye prevented infection. The tear film composition of rats is like humans with an outermost lipid layer and an innermost mucus layer, although rats do appear to lack a middle aqueous layer (22, 23), which could make rats less efficient at flushing out debris and toxins. One study found the fatty acids and alcohols secreted into the tear film by rats is compositionally similar to that of humans, although the abundance of some compounds differ (24). More efficient infection may occur if a corneal scratch method is used, as is done with herpesvirus infections (25, 26); however, we did not think that this was not a particularly relevant inoculation route for RVFV infection.

Using SC inoculation as an infection route, RVFV titers peaked in the eye at 3–4 dpi and posterior eye structures were infected, including the uvea, ciliary body, and retina. Damage or dysregulation of the uvea and ciliary body could impede the transport of nutrients and blood to both the anterior and posterior portion of the eye, contributing to pathology. Infection of the uvea and ciliary body may lead to a breach in the blood-retinal-barrier and blood-aqueous-barrier that these structures aid in maintaining (27, 28). This could result in an influx of inflammatory cells which may contribute to blinding ocular pathology (27, 29). At 3 days following SC infection, the ganglion layer of the retina and optic nerve were both infected, suggesting that the virus may use the optic nerve to gain access to the well-protected retina. Future studies should be performed to evaluate how RVFV traffics to the eye following SC infection.

Coinciding with the significant increase in chemokines, we observed an increase in general CD45^+^ leukocytic cells, including resident and peripheral myeloid cells, in the posterior region of eyes from RVFV-infected rats compared to eyes from uninfected rats. The increased presence of leukocytes in the posterior eye may be the result of the increased chemoattractants found in SC-infected rat eyes. CD45^+^CD11b^+^CD163^+^ cells have been described as M2 macrophages/monocytes that respond to anti-inflammatory signals such as IL-10, which is expressed at higher levels in SC-infected eyes (30, 31). The influx of leukocytes may exacerbate ocular pathology if the normal anti-inflammatory environment maintained by resident T_reg_ cells in the eye is overwhelmed (32). Additional studies are necessary to characterize the immune cell environment as well as inflammation in the eye at later time points.

Evaluation of the adaptive immune response in the eye may prove difficult due to the high lethality from RVFV infection in rats, but follow-up studies would be useful to determine its contribution to long-term ocular pathology. We noted high immune cell counts observed in some uninfected animals, which may be due to the development of spontaneous retinal lesions that can occasionally occur in Sprague Dawley rats (33). We were unable to complete in depth analysis of retinal function and health through fundus imaging or electroretinogram due to a lack of both accessibility and sensitivity of this equipment in the ABSL-3. Attenuated strains of RVFV including MP-12, Clone 13, DelNSs, and DelNSm should be tested in this rat model to determine if ocular tropism and pathology results from vaccine strain inoculation.

Based on the cell types that were infected in the rats, we tested human retinal pigment epithelial and uveal cell lines and found them to be highly permissive to infection by RVFV. We found cytokine responses secreted by ARPE-19 cells to be similar to the whole eye homogenate of the rat eyes, as secretion of IL-6, MCP-1 and GRO/KC was significantly increased following infection.

Lrp1 was recently identified as an essential host factor for RVFV infection in cells from taxonomically disparate species, and Lrp1 is necessary for lethal disease *in vivo* (19). Lrp1 also plays a role in efficient RVFV infection of human retinal pigment epithelial cells. Viral titers were significantly reduced following pre-treatment of ARPE-19 cells with the high-affinity Lrp1 ligand RAP. In humans, the macula and retinal pigment epithelial cells express high levels of Lrp1, and therefore could account for the high rate of retinal involvement in RVF ocular disease patients (34, 35).

Ocular complications are described in other arboviral infections, and this is most prominent with Zika virus (ZIKV) but also Dengue (DENV) and Chikungunya (CHIKV) viruses (36). Ocular manifestations in adult patients with acute Zika infection commonly involve the anterior segment of the eye (37). ZIKV viral RNA has been detected in the anterior ocular fluid, and treatment with steroids clear anterior uveitis and ZIKV RNA (37). Additionally, retinal pathology has been observed in infants with Zika virus microcephaly (38). Tractable mouse models have been developed to investigate the pathogenic mechanisms of Zika virus ocular disease (39, 40). Ocular manifestations are infrequent in Dengue fever; however, hyposphagma and rare cases of posterior segment involvement, including bilateral retinal hemorrhage and vasculitis, have been noted (36, 41). While some Dengue-infected individuals experience permanent vision deficiencies, most resolve without treatment (41, 42). CHIKV ocular manifestations are also considered rare, but retinal involvement and anterior uveitis have been described in CHIKV outbreaks (43).

Ocular infection and resulting vision problems are the most common complication of RVFV infection in people, and vision loss occurs in people with both mild and severe systemic disease (7, 8, 44). While vision problems present frequently in RVFV-infected people, the mechanisms regulating ocular infection have not been defined. Our study using Sprague Dawley rats presents a tractable *in vivo* model to understand the mechanisms of RVF ocular disease. Here, we show that despite eye infection not occurring in every infected rat, most posterior ocular structures, including the retina and optic nerve, are permissible to RVFV infection. We observed inflammation of eyes even in the absence of significant viral burden, as well as an increase in leukocyte counts in the posterior portion of the eye. Aside from gaining insight into pathogenesis, this model could be utilized for screening of vaccines for preventing RVF ocular disease and antivirals or other therapeutics for treating RVF ocular manifestations. Uncovering mechanisms of pathogenesis and identification of therapeutic targets could greatly benefit individuals affected by this debilitation ailment.

## MATERIALS AND METHODS

### Biosafety.

Work with wild-type ZH501 RVFV was conducted in the Regional Biocontainment Laboratory in the Center for Vaccine Research at the University of Pittsburgh, which is approved by the Federal Select Agent Program (FSAP) for work with virulent RVFV. All team members wore powered-air purifying respirators (PAPRs; 3M VersaFlo) when conducting work in the BSL-3 or ABSL-3. All surplus infectious material was inactivated in Vesphene IISE (1:128) for at least 10 min at room temperature.

### Viruses.

The wild-type ZH501 strain of Rift Valley fever virus was generously provided by Stuart Nichol (CDC, Atlanta, Georgia). All viruses were propagated in Vero E6 cells in Dulbecco’s modified Eagle’s medium (DMEM) with 2% fetal bovine serum (FBS), 1% penicillin-streptomycin (pen/strep), and 1% l-glutamine (hereby, D2). The titer of each virus was determined using a standard viral plaque assay (VPA) or TCID_50_ as described previously (12, 45).

### Cells.

ARPE-19 cells were kindly provided by Kip Kinchington (University of Pittsburgh, Pittsburgh, PA). Cells were maintained in DMEM/F12 media with 10% FBS. MP41 cells (ATCC, CRL-3297) were maintained in Eagles Modified Essential Medium with 20% FBS. BCE cells were kindly provided by Gaya Amarasinghe (WUSTL, St. Louis) and were maintained in DMEM with 10% FBS, 1% pen/strep, and 1% l-glutamine. Vero E6 cells (ATCC, CRL-1586) were used for all VPAs and were maintained in DMEM with 10% FBS, 1% pen/strep, and 1% l-glutamine.

### Animal experiments.

All animal work was approved by the University of Pittsburgh IACUC under protocol #20087344. Sprague Dawley rats were purchased from Envigo at approximately 8–10 weeks of age. Prior to infection, a temperature probe was inserted in the scruff behind the head (BMDS, IPT-300). Subcutaneous infection was performed by injecting 200 μL of RVFV diluted in D2 since components are already defined above into the right hind flank of the rat while it was anesthetized with isoflurane. RVFV ZH501 was concentrated using a 100 kDa cutoff Amicon column (EMD Millipore) for use in conjunctival inoculation. Live virus was quantified by VPA following concentration. Direct conjunctiva infection was conducted by pipetting 25 μL of RVFV diluted in D2 media (500 PFU into each eye for a total dose of 1000 PFU) underneath the left and right eyelids. The eyelids were opened and closed to ensure the complete volume was absorbed. The control group of rats also had conjunctiva exposure to D2 media.

For aerosol infection, rats were exposed in a whole-body chamber inside a class III biological safety cabinet to aerosols containing RVFV. All aerosol exposures were conducted using the Aero3G management platform (Biaera). All aerosols were conducted as dynamic aerosol exposures such that total air into and out of the chamber allowed for a complete air change in the chamber every 2 min. RVFV aerosols were generated with an Aerogen Solo vibrating mesh nebulizer (Aerogen). Based on prior RVFV aerosols, a humidification loop was added to the input air to ensure relative humidity was least 80%. Glycerol and antifoam were added to nebulizer and sampler media to optimize virus recovery. Total airflow into the chamber was set to 19.5 liters per minute (lpm). Aerosol samples were collected in an all-glass impinger (AGI-30, Ace Glass) connected to the exposure chamber and operated at 6 lpm. Total exhaust flow was set to 19.5 lpm. AGI samples were collected as previously described in cell culture media, and plaque assays were performed to determine aerosol concentration and inhaled dose. Plaque assays were also done on pre-nebulization contents to compare aerosol performance to prior aerosols.

Following infection, temperature and weight were monitored daily and scored based on change from baseline. If animals lost over 20% of body weight, or temperature was greater than 39.5°C or less than 34°C they were euthanized promptly. Animals were checked twice daily for clinical signs of illness including ruffled fur (1 point), hunching (2 points), porphyrin staining (3 points), and neurological signs such as circling (3 points), tremors (2 points) or loss of muscle coordination (1 point). In combination with weight and temperature scores, if a rat reached a score of 8, it was euthanized promptly. Additionally, if a rat was found recumbent or was experiencing seizures, it was euthanized immediately. Euthanasia was performed by isoflurane anesthesia followed by cervical dislocation. When eyes were to be used for histological analysis, anesthetized rats were perfused with saline followed by 4% paraformaldehyde (PFA). Timed euthanasia was conducted between 3 and 10 dpi, unless rats met IACUC-approved euthanasia criteria earlier based on clinical scoring. Eyes and other organs of interest were removed and stored for analysis.

### Immunofluorescence staining.

Eyes were fixed in 4% PFA for at least 24 h prior to transfer to 1XPBS. Following 24 h in 1XPBS, eyes were transferred to 15% sucrose overnight, followed by 30% sucrose overnight. Eyes in 30% sucrose were given to the Histology Core at the Ear and Eye Institute at the University of Pittsburgh for cryopreserving and sectioning. Eyes were cryosectioned at either 7 or 10 μm thickness and mounted onto 1.5 thickness silane coated positively charged glass slides (Azer Scientific). Proteins of interest were visualized in tissue sections using fluorescence IHC by permeabilizing with 0.1% TritonX100 in 1X PBS at room temperature for 15 min. Following 3 washes with 0.5% bovine serum albumin (BSA) in 1X PBS (PBB), slides were incubated with 5% normal donkey serum in PBB for 45 min at room temperature. After washing off the block 3 times with PBB, slides were incubated 1 h at room temperature with a rabbit anti-RVFV N antibody (custom made by Genscript) described in (46) diluted 1:100 in PBB. Following three washes with PBB, a donkey anti-rabbit 594 (JacksonImmuno, 711-585-152) secondary was diluted 1:1000 in PBB and slides were incubated for 1 h at room temperature. After washing 3 times with 1X PBS, slides were incubated with Hoescht (CBI) for 30 s. Slides were mounted with gelvatol. ICC staining followed the same protocol apart from blocking slides in 5% normal goat serum and the addition of anti-RVFV N (BEI, NR-43188), rabbit RVFV N at 1:200 (BEI, NR-43190), or rabbit anti Lrp1 1:200 (Abcam, ab92544) primary antibodies, and goat anti-rabbit 488 (JacksonImmuno, 111-545-003) and goat anti-mouse 594 (JacksonImmuno, 115-585-003) secondaries.

### Tissue or supernatant analysis.

Eye, brain, and liver tissue were homogenized and analyzed by viral plaque assay (12), or by q-RT-PCR for the L segment of RVFV (47). Cytokines and chemokines were detected in the whole eye homogenate using the Bio-Rad 23-plex rat inflammation panel (Bio-Rad, #12005641). Samples were diluted 1:2 and the manufacturer’s protocol was followed. IL-6, GRO/KC, and MCP-1 were detected in the supernatant of RVFV-infected ARPE-19 cells using the DuoSet ELISA (DY206-05, DY275-05, DY279-05) with undiluted sample.

### Flow Cytometry.

Right eyes were enucleated from euthanized rats at 3 dpi. The eye was stored in cold PBS until the posterior portion was dissected away from the cornea. The posterior eye tissue was digested in D10 + 1 mg/mL collagenase type II (Sigma-Aldrich, Cat# 234115) for 30 min at 37°C. Every 10 min, the tissue was triturated using a wide-mouth pipette for manual digestion. Following the 30-minute digestion, the sclera was removed and discarded. The cell suspension was run through a 40 μM filter and counted. We were able to consistently obtain 1x10^6^-1x10^7^ cells per eye. The single cell suspension was stained with LIVE/DEAD Fixable Blue kit (Thermo, L23105) on ice for 30 min followed by a block in 2% BSA in 1X PBS on ice for 30 min. Following 2 washes with 2% BSA in 1X PBS, the following antibodies were used for surface staining: CD45 AF700 (Bio-Rad, MCA43A700), CD11b (Bio-Rad, MCA619R), CD163 FITC (Bio-Rad, MCA342F). The cells were incubated with these antibodies for 30 min on ice, followed by 2 washes with 1X PBS in 2% BSA. The cells were fixed and permeabilized with the BD Cytofix/Cytoperm Kit (BDsciences, 554714), for 20 min on ice. Following 2 washes with the BDsciences Fix/Perm wash, the cells were stained with RVFV N (custom, Genescript) for 30 min on ice. The cells were washed 2 times with 1X PBS in 2% BSA and stained with donkey anti-rabbit 594 (JacksonImmuno, 711-585-152) for 20 min on ice. The cells were washed 3 times with 1X PBS in 2% BSA and fixed in 4% PFA for 15 min for evaluation outside of containment. Compensation was preformed using the AbC Total Antibody Compensation Bead kit (Thermo, A10497), and the samples were run on a LSRII Fortessa acquiring over 5x10^5^ cells and gated using the strategy described in Fig. S2. All analysis was performed in FlowJo v10.

### Statistics.

All statistics were calculated through GraphPad Prism v9. A one-way ANOVA was used to determine significant differences between more than 2 experimental groups. An *unpaired t test* was used to determine significant differences between 2 groups. Error bars represent standard error mean. *, *P* < 0.05; **, *P* < 0.01; ***, *P* < 0.001.
