# Supplementary material for: Rift Valley Fever Virus Infects the Posterior Segment of the Eye and Induces Inflammation in a Rat Model of Ocular Disease
Source: J Virol. 2022 Oct 4;96(20):e01112-22. doi: 10.1128/jvi.01112-22 (PMC9599513; doi:10.1128/jvi.01112-22)
Supplement: Supplemental file 1 — Fig. S1 and S2. Download jvi.01112-22-s0001.pdf, PDF file, 0.9 MB [file jvi.01112-22-s0001.pdf]

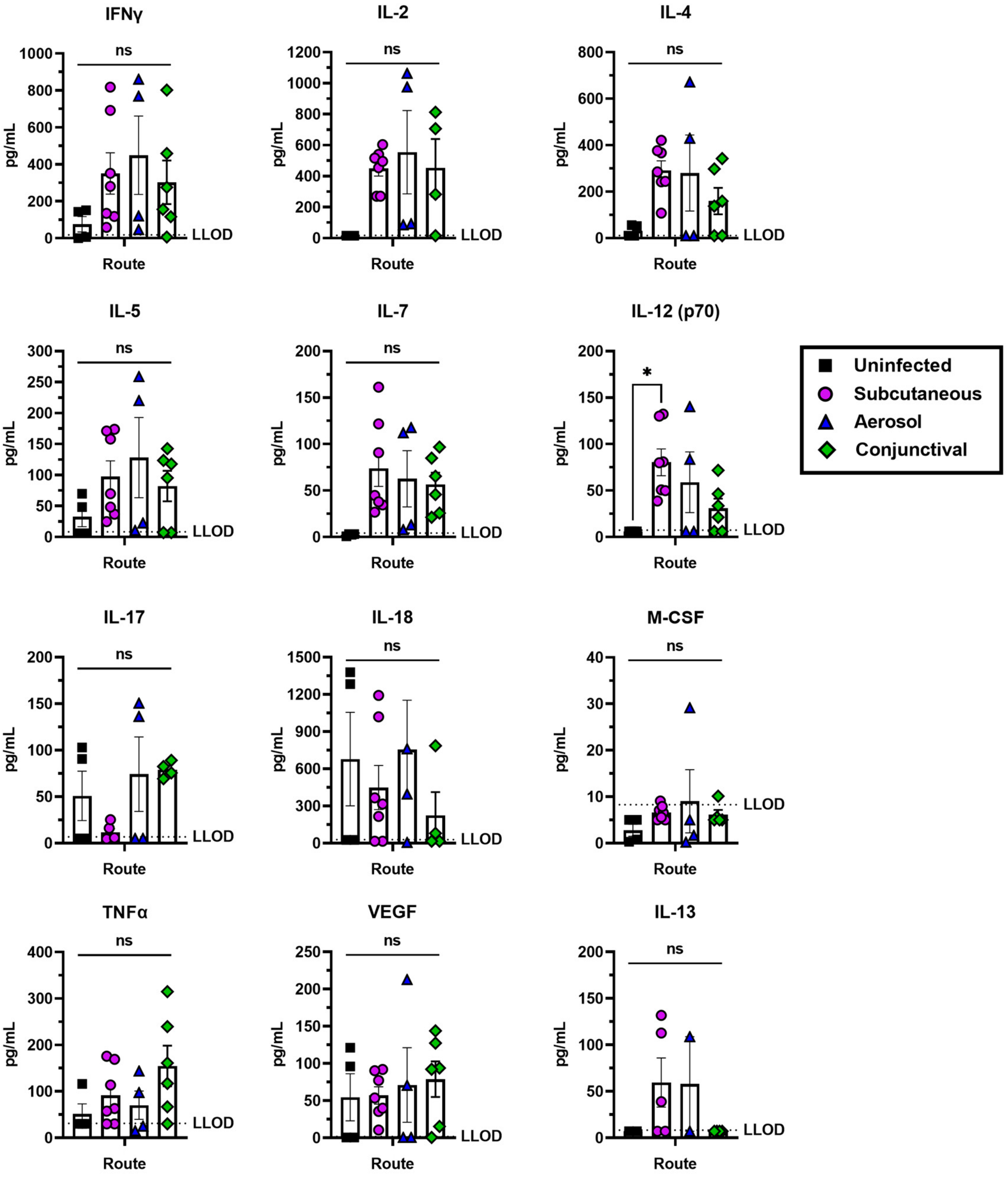

Figure S1: Inflammatory factors in the eye at 4 dpi with RVFV ZH501. Additional inflammatory factors analyzed in whole eye homogenate by multiplex assay. Rats were infected through sham (uninfected), subcutaneous, aerosol, or conjunctival inoculation. Each datapoint represents one eye from one rat across 4 experiments. Error bars are standard error mean. LLOD = Lower limit of detection. *, $p<0.05$. Ns = not significant.

Figure S1

A

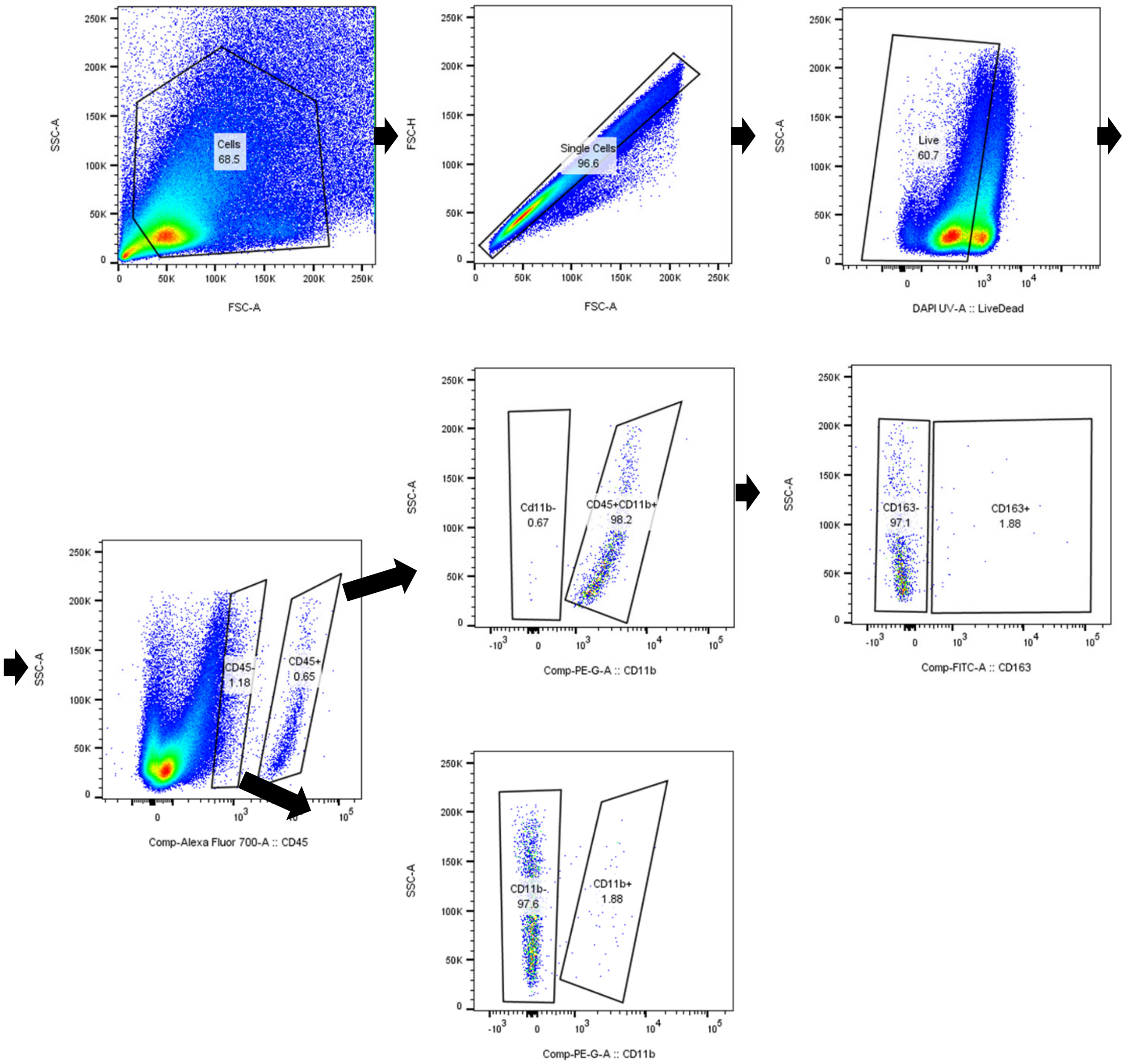

Figure S2: Gating strategy for flow cytometry on posterior ocular cells. Representative 3 dpi posterior eye sample from a rat infected SC with RVFV ZH501. (A) 500,000 cells were collected per sample. Singlets were gated upon, followed by gating on live cells. CD45 positive and negative cells were gated on followed by CD11b positive and negative populations. From CD45 positive and CD11b positive cells, CD163 positive and negative cells were gated for.

Figure S2
